# Soft Tissue Infection of the Upper Extremity

**Published:** 2011-04-11

**Authors:** Rajiv P. Parikh, Effie Pappas-Politis

**Affiliations:** Division of Plastic Surgery, University of South Florida, Tampa

## DESCRIPTION

A 54-year-old man with severe left upper extremity pain presented to the emergency department 1 day after a minor abrasion to the forearm. Physical examination of the extremity revealed warmth, erythema, exquisite tenderness, blue/violet skin discoloration, and tense bullae containing serous fluid. Systemically, the patient was hypotensive, tachycardic, febrile, and had a white blood cell count of 16500. A “finger test” was positive.

## QUESTIONS

1. **What is the diagnosis?**
2. **What is the most likely infectious etiology?**
3. **How is the diagnosis made and what is the significance of the “finger test”?**
4. **What is the treatment strategy?**

## DISCUSSION

Necrotizing fasciitis is a rare, but life-threatening, soft-tissue infection. First coined by Wilson in 1952, the term refers to a necrotizing infectious process that rapidly progresses along fascial layers within the soft-tissue compartment.^1^ Necrotizing fasciitis is classified into 2 types to reflect its bacterial etiology.^2^ Type I infections are more prevalent, usually occur after trauma or surgery, and have a polymicrobial etiology. Most commonly, this is non–group A streptococci in synergism with other aerobe and anaerobe organisms.^2^^-^5 Type II infections are monomicrobial and often develop without an antecedent trauma. Group A, B-hemolytic streptococci, and *Staphylococcus aureus* are the most common pathogens in these cases.^2^^-^5

Despite the different organisms responsible, both types of necrotizing fasciitis share a similarly devastating pathophysiology. Microbial proliferation causes inflammation, liqeufactive necrosis of the fascial layers, and eventual thrombosis of the perforating vessels. The result is rapidly expanding ischemia with subsequent gangrene of the skin, subcutaneous fat, fascia, and skeletal muscle. As the course evolves from a local to a regional to a fulminant process, systemic inflammation, shock, and multiorgan failure become the terminal endpoints.^1^^-^10

To reduce morbidity and mortality, it is critical to make an early clinical diagnosis.^4^^-^8 Patients usually present reporting pain; however, clinical findings may initially be subtle with skin changes of only erythema and swelling.^4^^-^6 Differentiating necrotizing fasciitis from less-aggressive soft-tissue infections that share this initial presentation is of the utmost importance. Frequent repeat physical examinations are needed to evaluate rapid progression. The development of pain out of proportion to physical examination, subcutaneous tissue hardening, and tense edema outside areas of erythema distinguish this condition from simpler infections.^4^^-^6,9 Elevated white blood cells counts more than 15400 cells/mm^3^, serum Na^+^ less than 135 mmol/L, c-reactive protein more than 150 mg/L, creatinine more than 1.6 mg/dL, glucose more than 180 mg/dL, and/or hemoglobin less than 11 g/dL are laboratory variables that may aid in discriminating necrotizing from nonnecrotizing infections.^9^,^10^ Additional roles for plain film, ultrasonography computed tomography, and magnetic resonance imaging have been identified. However, these tests should never delay intervention or precede clinical examination. Ultimately, systemic findings of fever, tachycardia, or hypotension, accompanying local findings of bullae, crepitus, skin necrosis or sensorimotor defects, are enough to mandate operative intervention.

The first step in this intervention is the “finger test.”^4^,^5^ A small incision extending down to the deep fascia is made in the involved area. A finger is then used to gently dissect tissue at the deep fascial level. A positive test is characterized by minimal tissue resistance to finger dissection. A positive finger test, the absence of bleeding, presence of necrotic tissue, and/or presence of murky, grayish (“dishwater”) fluid following incision all support the diagnosis of a sinister necrotizing process (Figure 1). In these cases, the incision can be promptly extended to begin emergency surgery.

The treatment strategy should always include extensive surgical debridement (Figure 2) with appropriate broad-spectrum antibiotic coverage. All compromised tissues must be operatively removed. Frequent reevaluation of the wound and repetitive debridement with washings is necessary until healthy granulation tissue is present.^4^^-^8 Only then can reconstructive efforts commence to close or cover defects. Of interest, hyperbaric oxygen is part of the treatment approach at several institutions. There is evidence, albeit from small case studies, to support this therapy as an adjunct to, not as a replacement of, the standard protocol.^8^

## Figures and Tables

**Figure:**
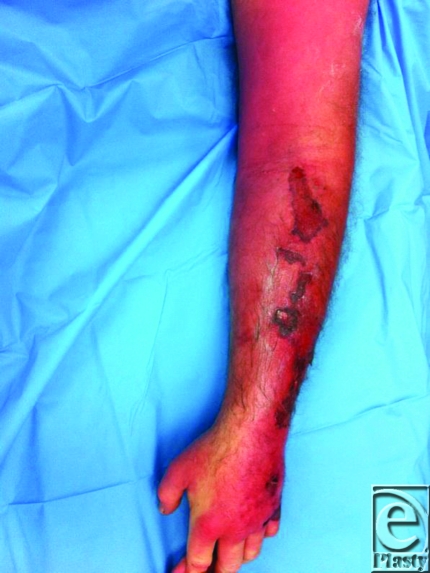


**Figure 1:**
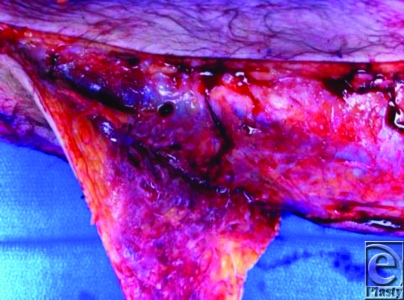
Necrotic tissue and “dishwater” fluid with a positive finger test.

**Figure 2:**
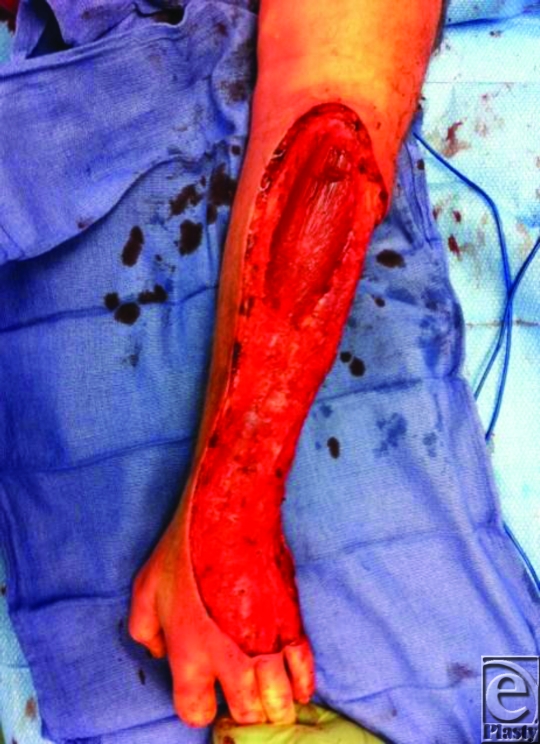
Treatment: Extensive debridement of devitalized tissue.
